# Early Estimate of Nirsevimab Effectiveness for Prevention of Respiratory Syncytial Virus–Associated Hospitalization Among Infants Entering Their First Respiratory Syncytial Virus Season — New Vaccine Surveillance Network, October 2023–February 2024

**DOI:** 10.15585/mmwr.mm7309a4

**Published:** 2024-03-07

**Authors:** Heidi L. Moline, Ayzsa Tannis, Ariana P. Toepfer, John V. Williams, Julie A. Boom, Janet A. Englund, Natasha B. Halasa, Mary Allen Staat, Geoffrey A. Weinberg, Rangaraj Selvarangan, Marian G. Michaels, Leila C. Sahni, Eileen J. Klein, Laura S. Stewart, Elizabeth P. Schlaudecker, Peter G. Szilagyi, Jennifer E. Schuster, Leah Goldstein, Samar Musa, Pedro A. Piedra, Danielle M. Zerr, Kristina A. Betters, Chelsea Rohlfs, Christina Albertin, Dithi Banerjee, Erin R. McKeever, Casey Kalman, Benjamin R. Clopper, New Vaccine Surveillance Network Product Effectiveness Collaborators, Meredith L. McMorrow, Fatimah S. Dawood, New Vaccine Surveillance Network Product Effectiveness Collaborators, Ruth Link-Gelles, Amanda Payne, Ryan Wiegand, Ximena Aguilera Correa, Claudia Guevara Pulido, Hanna Grioni, Bonnie Strelitz, Vasanthi Avadhanula, Flor M. Munoz, Wende Fregoe, Saranya Peri, Anjana Sasidharan, Monika Johnson, Klancie Dauer

**Affiliations:** ^1^Coronavirus and Other Respiratory Viruses Division, National Center for Immunization and Respiratory Diseases, CDC; ^2^UPMC Children’s Hospital of Pittsburgh, Pittsburgh, Pennsylvania; ^3^Department of Pediatrics, University of Pittsburgh School of Medicine, Pittsburgh, Pennsylvania; ^4^Texas Children’s Hospital, Houston, Texas; ^5^Baylor College of Medicine, Houston, Texas; ^6^Department of Pediatrics, Seattle Children’s Hospital, Seattle, Washington; ^7^Department of Pediatrics, Vanderbilt University Medical Center, Nashville, Tennessee; ^8^Division of Infectious Diseases, Cincinnati Children’s Hospital Medical Center, Cincinnati, Ohio; ^9^Department of Pediatrics, University of Cincinnati College of Medicine, Cincinnati, Ohio; ^10^Department of Pediatrics, University of Rochester Medical Center and University of Rochester–Golisano Children’s Hospital, Rochester, New York; ^11^Department of Pathology and Laboratory Medicine, Children’s Mercy Hospital, Kansas City, Missouri; ^12^Department of Pediatrics Children’s Mercy Hospital, Kansas City, Missouri.; Coronavirus and Other Respiratory Viruses Division, National Center for Immunization and Respiratory Diseases, CDC; Coronavirus and Other Respiratory Viruses Division, National Center for Immunization and Respiratory Diseases, CDC; Coronavirus and Other Respiratory Viruses Division, National Center for Immunization and Respiratory Diseases, CDC; Department of Pediatrics, Vanderbilt University Medical Center; Department of Pediatrics, Vanderbilt University Medical Center; Department of Pediatrics, Seattle Children’s Hospital; Department of Pediatrics, Seattle Children’s Hospital; Baylor College of Medicine; Texas Children’s Hospital and Baylor College of Medicine; Department of Pediatrics, University of Rochester Medical Center and University of Rochester–Golisano Children’s Hospital; Department of Pathology and Laboratory Medicine, Children’s Mercy Kansas City; Department of Pathology and Laboratory Medicine, Children’s Mercy Kansas City; Department of Pediatrics, University of Pittsburgh School of Medicine; Department of Pediatrics, University of Pittsburgh School of Medicine

**Summary**

**What is already known about this topic?**

Respiratory syncytial virus (RSV) is the leading cause of hospitalization among U.S. infants. In August 2023, CDC recommended nirsevimab, a long-acting monoclonal antibody, to protect infants aged <8 months against RSV-associated lower respiratory tract infection in their first RSV season.

**What is added by this report?**

Nirsevimab effectiveness was 90% against RSV-associated hospitalization in infants in their first RSV season. Median time from receipt of nirsevimab to symptom onset was 45 days (IQR = 19–76).

**What are the implications for public health practice?**

To reduce the risk for RSV-associated hospitalization, infants should be protected by maternal RSV vaccination or infant receipt of nirsevimab.

## Abstract

Respiratory syncytial virus (RSV) is the leading cause of hospitalization among infants in the United States. In August 2023, CDC’s Advisory Committee on Immunization Practices recommended nirsevimab, a long-acting monoclonal antibody, for infants aged <8 months to protect against RSV-associated lower respiratory tract infection during their first RSV season and for children aged 8–19 months at increased risk for severe RSV disease. In phase 3 clinical trials, nirsevimab efficacy against RSV-associated lower respiratory tract infection with hospitalization was 81% (95% CI = 62%–90%) through 150 days after receipt; post-introduction effectiveness has not been assessed in the United States. In this analysis, the New Vaccine Surveillance Network evaluated nirsevimab effectiveness against RSV-associated hospitalization among infants in their first RSV season during October 1, 2023–February 29, 2024. Among 699 infants hospitalized with acute respiratory illness, 59 (8%) received nirsevimab ≥7 days before symptom onset. Nirsevimab effectiveness was 90% (95% CI = 75%–96%) against RSV-associated hospitalization with a median time from receipt to symptom onset of 45 days (IQR = 19–76 days). The number of infants who received nirsevimab was too low to stratify by duration from receipt; however, nirsevimab effectiveness is expected to decrease with increasing time after receipt because of antibody decay. Although nirsevimab uptake and the interval from receipt of nirsevimab were limited in this analysis, this early estimate supports the current nirsevimab recommendation for the prevention of severe RSV disease in infants. Infants should be protected by maternal RSV vaccination or infant receipt of nirsevimab.

## Introduction

Respiratory syncytial virus (RSV) is the leading cause of hospitalization in U.S. infants, responsible for 50,000–80,000 hospitalizations annually in children aged <5 years (*1*,*2*). The highest hospitalization rates occur during the first months of life, and risk declines with increasing age in infancy and during early childhood (*3*). In August 2023, CDC’s Advisory Committee on Immunization Practices (ACIP) recommended nirsevimab, a long-acting monoclonal antibody, for all infants aged <8 months born during or entering their first RSV season, and for children aged 8–19 months at increased risk for severe RSV disease and entering their second RSV season (*4*). In a pooled analysis of data from prelicensure randomized placebo-controlled clinical trials, 1 dose of nirsevimab given at age <8 months was 79% efficacious against medically attended RSV-associated lower respiratory tract infection and 81% efficacious against RSV-associated lower respiratory tract infection with hospitalization through 150 days after injection (*4*). In September 2023, a maternal RSV vaccine also became available to prevent RSV disease in young infants. ACIP recommends either nirsevimab or maternal RSV vaccination to protect infants born during or entering their first RSV season (*5*). In October 2023, in response to nirsevimab shortages, CDC recommended that health care settings with limited supply of nirsevimab prioritize nirsevimab for infants aged <6 months and infants with underlying conditions at highest risk for severe disease (*6*). In January 2024, additional doses of nirsevimab became available, and CDC recommended that health care settings with adequate nirsevimab supply return to the original ACIP recommendations for nirsevimab use (*7*). This analysis provides the first U.S. estimate for post-introduction nirsevimab effectiveness among U.S. infants during their first RSV season.

## Methods

### Data Collection and Inclusion Criteria

The New Vaccine Surveillance Network (NVSN) is a population-based, prospective surveillance platform for acute respiratory illness (ARI) in infants, children, and adolescents aged <18 years that monitors pediatric respiratory viruses at seven U.S. pediatric academic medical centers to assess immunization effectiveness.^†^ Demographic, clinical, and immunization data were systematically collected through parent/guardian interviews, medical record abstraction, and state immunization information systems. Respiratory specimens were collected from enrolled children and tested for RSV and other common respiratory viruses by real-time reverse transcription–polymerase chain reaction.^§^ Receipt of nirsevimab was ascertained through parent report and verified through state immunization information systems, birth hospital, or primary care provider records.^¶^

Infants were eligible for this analysis if they were aged <8 months as of October 1, 2023, or born after October 1, 2023, were hospitalized with ARI** during October 1, 2023–February 29, 2024, and had verified nirsevimab status, reported gestational age at birth, and medical record review to assess for underlying medical conditions. Infants were excluded if they were enrolled before nirsevimab became available at their site,^††^ received any doses of palivizumab, had reported maternal RSV vaccination during pregnancy, or inconclusive or unknown RSV test results. For a site to be included in this analysis, at least five infants enrolled at the site had to have received nirsevimab ≥7 days before symptom onset.

### Data Analysis

Nirsevimab effectiveness against RSV-associated hospitalization was estimated using a test-negative, case-control design. Case-patients were infants who received a positive RSV test result. Control patients were infants who received a negative RSV test result. Infants were considered nirsevimab recipients if they received nirsevimab ≥7 days before symptom onset to account for RSV incubation period and time to peak antibody concentration.^§§^ Infants who received nirsevimab <7 days before symptom onset were excluded. Pearson’s chi-square tests were used to compare demographic characteristics among case-patients and control patients and by nirsevimab status. Effectiveness was estimated using multivariable logistic regression models, comparing the odds of receipt of nirsevimab among case-patients and control patients. Regression models controlled for age at enrollment in months, month of illness, enrollment site, and presence of one or more high-risk medical conditions for severe RSV disease.^¶¶^ Preterm status (birth at <28, 28–31, 32–33, 34–36, and ≥37 weeks’ gestation) and insurance type were evaluated as potential confounders but did not change estimates and were not included in the final model. Effectiveness was calculated as (1− adjusted odds ratio) × 100%. Analyses were conducted using SAS software (version 9.4; SAS Institute). This activity was reviewed by CDC, deemed not research, and was conducted consistent with applicable federal law and CDC policy.***

## Results

Among 1,036 eligible infants, 699 infants at four sites met inclusion criteria,^†††^ including 407 (58%) case-patients and 292 (42%) control patients (Table). Receipt of nirsevimab was more frequent among infants with high-risk medical conditions than those without these conditions (46% versus 6%, p<0.001). There was no difference in the frequency of receipt of nirsevimab by preterm status or insurance type. Time since receipt of nirsevimab to ARI symptom onset ranged from 7 to 127 days with a median of 45 days (IQR = 19–76 days) (Figure). Overall, six (1%) case-patients and 53 (18%) control patients received nirsevimab; among all included infants, receipt of nirsevimab ranged from 4% to 12% by site. Nirsevimab effectiveness was 90% (95% CI = 75–96) against RSV-associated hospitalization.

**TABLE:** Characteristics of infants born during or entering their first respiratory syncytial virus season who were hospitalized with acute respiratory illness, by respiratory syncytial virus test result and receipt of nirsevimab*^,^^†^ — New Vaccine Surveillance Network, October 2023–February 2024

Characteristic	Overall total, no. (column %)	RSV test result			Receipt of nirsevimab		
		Positive no. (column %)	Negative no. (column %)	p-value^§^	Yes no. (row %)	No no. (row %)	p-value^§^
**All children**	**699**	**407 (58)**	**292 (42)**	**—**	**59 (8)**	**640 (92)**	**—**
**Age group at admission, mos**							
<1	111 (16)	51 (13)	60 (21)	<0.001	10 (9)	101 (91)	0.028
1–2	214 (31)	144 (35)	70 (24)		18 (8)	196 (92)	
3–4	131 (19)	90 (22)	41 (14)		9 (7)	122 (93)	
5–6	121 (17)	67 (16)	54 (18)		6 (5)	115 (95)	
7–8	96 (14)	49 (12)	47 (16)		9 (9)	87 (91)	
9–10	23 (3)	6 (1)	17 (6)		6 (26)	17 (74)	
11–12	3 (0)	0 (—)	3 (1)		1 (33)	2 (67)	
**Gestational age**							
Preterm (<37 wks)^¶^	146 (21)	77 (19)	69 (24)	0.129	15 (10)	131 (90)	0.377
Term (≥37 wks)	551 (79)	329 (81)	222 (76)		44 (8)	507 (92)	
Unknown	2 (0)	1 (0)	1 (0)		0 (—)	2 (100)	
High-risk medical condition**							
None	660 (94)	396 (97)	264 (90)	<0.001	41 (6)	619 (94)	<0.001
≥1	39 (6)	11 (3)	28 (10)		18 (46)	21 (54)	
**Sex**							
Female	293 (42)	182 (45)	111 (38)	0.076	28 (10)	265 (90)	0.367
Male	406 (58)	225 (55)	181 (62)		31 (8)	375 (92)	
**Race and ethnicity^††^**							
American Indian or Alaska Native	1 (0)	1 (0)	0 (—)	0.002	0 (—)	1 (100)	0.511
Asian	47 (7)	27 (7)	20 (7)		3 (6)	44 (94)	
Black or African American	89 (13)	41 (10)	48 (16)		8 (9)	81 (91)	
Native Hawaiian or other Pacific Islander	225 (32)	126 (31)	99 (34)		23 (10)	202 (90)	
White	30 (4)	12 (3)	18 (6)		5 (17)	25 (83)	
Hispanic or Latino	8 (1)	3 (1)	5 (2)		0 (—)	8 (100)	
Multiple race or other non-specified	280 (40)	188 (46)	92 (32)		18 (6)	262 (94)	
Unknown	19 (3)	9 (2)	10 (3)		2 (11)	17 (89)	
**Insurance status**							
Public	385 (55)	198 (49)	187 (64)	<0.001	37 (10)	348 (90)	0.296
Private	233 (33)	155 (38)	78 (27)		17 (7)	216 (93)	
Public and private	4 (1)	2 (0)	2 (1)		1 (25)	3 (75)	
Self-pay (none)	51 (7)	31 (8)	20 (7)		4 (8)	47 (92)	
Unknown	26 (4)	21 (5)	5 (2)		0 (—)	26 (100)	
**Site**							
Houston, TX	195 (28)	110 (27)	85 (29)	0.050	24 (12)	171 (88)	0.013
Nashville, TN	93 (13)	47 (12)	46 (16)		9 (10)	84 (90)	
Pittsburgh, PA	235 (34)	153 (38)	82 (28)		9 (4)	226 (96)	
Seattle, WA	176 (25)	97 (24)	79 (27)		17 (10)	159 (90)	
**RSV test result**							
Positive	407 (58)	NA	NA	—	6 (1)	401 (99)	<0.001
Negative	292 (42)	NA	NA		53 (18)	239 (82)	

**Abbreviations:** BPAP = bilevel positive airway pressure; CPAP = continuous positive airway pressure; NA = not applicable; RSV = respiratory syncytial virus.

* Overall, 337 infants enrolled during the analysis period were excluded. Reasons for exclusion included enrollment at sites with fewer than five infants who had received nirsevimab (296 from Rochester, Cincinnati, and Kansas City), receipt of nirsevimab <7 days before symptom onset (20), missing or inconclusive RSV test result (20), maternal receipt of RSV vaccine during pregnancy (22), and receipt of palivizumab (10); reasons for exclusion are not mutually exclusive.

^†^ Current season receipt of nirsevimab documented by registry or provider (654: 94%) or medical record only (45: 6%).

^§^ Pearson’s chi-square tests were used to compare demographic characteristics among case-patients and control patients and by receipt of nirsevimab.

^¶^ <28 weeks (12: 2%); 28–31 weeks (12: 2%); 32–33 weeks (48: 7%); 34–36 weeks (74: 11%).

** High-risk medical conditions were defined as chronic lung disease of prematurity (bronchopulmonary dysplasia, bronchiolitis obliterans, chronic respiratory failure with CPAP/BIPAP/ventilator, pulmonary hypertension [neonatal, primary, or secondary], or interstitial lung disease) (12); hemodynamically significant congenital heart disease (abnormalities of aortic arch, hypoplastic left heart syndrome, pulmonary atresia, tricuspid atresia, Tetralogy of Fallot, transposition of the great arteries, partial or total anomalous pulmonary venous return, other abnormalities of heart valves, double outlet right ventricle, or other congenital heart malformations) (21); severe immunocompromise (one); severe cystic fibrosis (two); neuromuscular disease (autonomic dysfunction, instability or dysautonomia, agenesis or hypoplasia of the corpus callosum, muscular dystrophy or spinal muscular atrophy, disorders of tone, or other neuromuscular condition) (12); or congenital pulmonary abnormalities that impair the ability to clear secretions (none).

^††^ Persons of Hispanic or Latino (Hispanic) origin might be of any race but are categorized as Hispanic; all racial groups are non-Hispanic.

**FIGURE:**
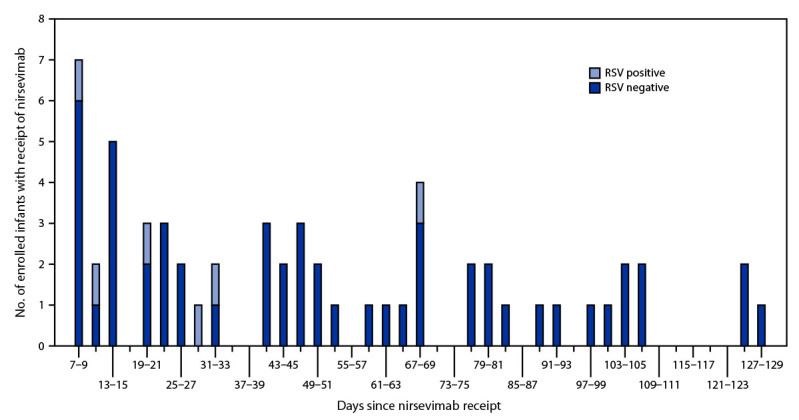
Time from receipt of nirsevimab* to symptom onset among infants born during or entering their first respiratory syncytial virus season who were hospitalized with acute respiratory illness, by respiratory syncytial virus test result — New Vaccine Surveillance Network, October 2023–February 2024 **Abbreviation:** RSV = respiratory syncytial virus. * Days 0–6 are not included because infants with receipt of nirsevimab within 7 days of symptom onset were excluded from this analysis.

## Discussion

In this multisite analysis of 699 infants hospitalized with ARI during their first RSV season, receipt of nirsevimab was 90% effective against RSV-associated hospitalization at a median of 45 days from receipt of nirsevimab to ARI symptom onset. This early effectiveness estimate supports existing recommendations for the prevention of severe RSV disease in infants in their first RSV season.

The strengths of this first estimate of U.S. post-introduction nirsevimab effectiveness include enrollment of infants using a standardized ARI definition, systematic RSV testing, and receipt of nirsevimab verification with state immunization information systems or medical records for all infants. However, it is important to note that nirsevimab effectiveness during a full RSV season is expected to be lower than the estimate reported here, because antibody levels from passive immunization wane over time. In this analysis, the median interval from receipt of nirsevimab was 45 days, whereas the median duration of the U.S. RSV season before the COVID-19 pandemic was 189 days (*8*). In clinical trials, nirsevimab remained highly efficacious against RSV-associated lower respiratory tract infection in infants through 150 days after receipt of nirsevimab, consistent with an extended half-life of 63–73 days (*9*).

Estimating effectiveness under real-world conditions for the full duration of an RSV season and in children aged 8–19 months at high risk for severe RSV disease who are recommended to receive nirsevimab before their second RSV season remains important. Thus, CDC will continue to monitor nirsevimab effectiveness.

### Limitations

The findings in this report are subject to at least five limitations. First, only a small proportion of hospitalized infants with ARI received nirsevimab, likely in part because of delayed availability in this first season of introduction and intermittent supply shortages, and infants who received nirsevimab were more likely to have underlying conditions.^§§§^ Thus, results might not be fully generalizable to all infants eligible for receipt of nirsevimab in their first RSV season. Second, the low number of case-patients who received nirsevimab did not allow for stratified estimates by time since receipt of nirsevimab. Third, because nirsevimab became available at most sites in the United States after seasonal RSV circulation began, some infants in this analysis might have had RSV infection before receipt of nirsevimab, which might have affected estimated effectiveness. Fourth, nirsevimab effectiveness was not estimated by dosage (50 mg for infants weighing <5 kg or 100 mg for infants weighing ≥5 kg) because nirsevimab dosage was not ascertained. Finally, the effectiveness estimate in this report is limited to the prevention of RSV-associated hospitalization. RSV among infants also causes a considerable increase in outpatient and emergency department visits; additional studies are warranted to assess nirsevimab effectiveness against these outcomes.

### Implications for Public Health Practice

Receipt of a single dose of nirsevimab was highly effective against RSV-associated hospitalization in infants entering their first RSV season. This finding supports current CDC recommendations that all infants should be protected by maternal RSV vaccination or infant receipt of nirsevimab, to reduce the risk for RSV-associated hospitalization in their first RSV season (*4*,*6*).

## Footnotes

* These senior authors contributed equally to this report.

† Children’s Mercy Hospital, Kansas City, Missouri; Cincinnati Children’s Hospital Medical Center, Cincinnati, Ohio; Golisano Children’s Hospital, Rochester, New York; Seattle Children’s Hospital, Seattle, Washington; Texas Children’s Hospital, Houston, Texas; UPMC Children’s Hospital of Pittsburgh, Pittsburgh, Pennsylvania; Vanderbilt University Medical Center, Nashville, Tennessee.

§ All enrolled children are tested for the following viruses: adenoviruses, SARS-CoV-2, rhinovirus/enterovirus, RSV, human metapneumovirus, enterovirus-D68, parainfluenza viruses, human coronaviruses, and influenza viruses.

¶ Primary care provider record verification was performed in sites without mandatory reporting of nirsevimab administration to state immunization information systems.

** ARI is defined as one or more of the following signs or symptoms present for <14 days before enrollment encounter: fever, cough, earache, nasal congestion, runny nose, sore throat, vomiting after coughing, wheezing, shortness of breath, rapid or shallow breathing, apnea, apparent life-threatening event, or brief resolved unexplained event.

†† 2023: Houston, Texas, October 5; Nashville, Tennessee, October 8; Seattle, Washington, October 8; Cincinnati, Ohio, October 10; Kansas City, Missouri, November 1; Pittsburgh, Pennsylvania, November 2; Rochester, New York November 6.

§§ In clinical trials, peak neutralizing antibody concentration levels were reached in adults by day 6 after intramuscular administration. https://www.accessdata.fda.gov/drugsatfda_docs/label/2023/761328s000lbl.pdf

¶¶ High-risk medical conditions were defined as chronic lung disease of prematurity (bronchopulmonary dysplasia, bronchiolitis obliterans, chronic respiratory failure with continuous positive airway pressure/bilevel positive airway pressure/ventilator, pulmonary hypertension, or interstitial lung disease) (11); hemodynamically significant congenital heart disease (abnormalities of aortic arch, hypoplastic left heart syndrome, pulmonary atresia, tricuspid atresia, Tetralogy of Fallot, transposition of the great arteries, partial or total anomalous pulmonary venous return, other abnormalities of heart valves, double outlet right ventricle, or other severe congenital heart malformations) (21); severe immunocompromise (one); severe cystic fibrosis (two); neuromuscular disease (autonomic dysfunction, instability or dysautonomia, agenesis or hypoplasia of the corpus callosum, muscular dystrophy or spinal muscular atrophy, disorders of tone, or other neuromuscular condition) (11); or congenital pulmonary abnormalities that impair the ability to clear secretions (none).

*** 45 C.F.R. part 46, 21 C.F.R. part 56; 42 U.S.C. Sect. 241(d); 5 U.S.C. Sect. 552a; 44 U.S.C. Sect. 3501 et seq.

††† Among the 337 infants excluded from this analysis, reasons for exclusion included enrollment at a site with fewer than five infants who had received nirsevimab (296 from Rochester, Cincinnati, and Kansas City), receipt of nirsevimab <7 days before symptom onset (20), missing or inconclusive RSV test result (20), maternal receipt of RSV vaccine during pregnancy (22), and receipt of palivizumab (10); reasons for exclusion are not mutually exclusive.

§§§ https://www.cdc.gov/vaccines/imz-managers/coverage/rsvvaxview/index.html (Accessed January 30, 2024).
